# Oncolytic adenovirus coding for shedding-resistant MICA enhances immune responses against tumors

**DOI:** 10.1007/s00262-023-03611-3

**Published:** 2024-01-05

**Authors:** M Costa-Garcia, JJ Rojas, MD Ramos, P Barlabé, P Calvo, J Navas, R Alemany, R Moreno

**Affiliations:** 1 Cancer Immunotherapy Group, Oncobell and iProCURE programs, IDIBELL-Institut Català d’Oncologia, l’Hospitalet de Llobregat, Barcelona, Spain; 2 Immunology Unit, Department of Pathology and Experimental Therapies, School of Medicine, University of Barcelona-UB, Barcelona, Spain; 3 Immunity, Inflammation, and Cancer Group, Oncobell program, Institut d’Investigació Biomèdica de Bellvitge-IDIBELL, l’Hospitalet de Llobregat, Barcelona, Spain; 4 Program of Regenerative Medicine, Centre for Applied Medical Research (CIMA), University of Navarra, Instituto de Investigación Sanitaria de Navarra (IdiSNA), Pamplona, Pamplona, 31008 Spain

**Keywords:** Oncolytic adenovirus, Cancer, Immunotherapy, NK cells, MICA

## Abstract

Cancer immunotherapies strive to overcome tumor-induced immune suppression and activate antitumor immune responses. Although cytotoxic T lymphocytes (CTLs) play a pivotal role in this process, natural killer (NK) cells have also demonstrated remarkable tumor-killing abilities, given their ability to discriminate tumor cells from normal cells and mediate specific antitumoral cytotoxicity. NK cells activation depends on a balance between activation and inhibition signals from several ligands/receptors. Among them, MICA/NKG2D axis is a master regulator of NK activation. MHC class I chain-related polypeptide A (MICA) expression is upregulated by many tumor cell lines and primary tumors and serves as a ligand for the activating NK group 2D (NKG2D) receptor on NK cells and subpopulations of T cells. However, cancer cells can cleave MICA, making it soluble and de-targeting tumor cells from NK cells, leading to tumor immune escape.

In this study, we present ICOVIR15KK-MICAMut, an oncolytic adenovirus (OAdv) armed with a transgene encoding a non-cleavable MICA to promote NK-mediated cell-killing capacity and activate the immune response against cancer cells. We first demonstrated the correct MICA overexpression from infected cells. Moreover, our MICA-expressing OAdv promotes higher NK activation and killing capacity than the non-armed virus in vitro. In addition, the armed virus also demonstrated significant antitumor activity in immunodeficient mice in the presence of human PBMCs, indicating the activation of human NK cells. Finally, OAdv-MICA overexpression in immunocompetent tumor-bearing mice elicits tumor-specific immune response resulting in a greater tumor growth control.

In summary, this study highlights the significance of NK cells in cancer immunotherapy and presents an innovative approach using a modified oncolytic virus to enhance NK cell activation and antitumor immune response. These findings suggest promising potential for future research and clinical applications.

**Supplementary Information:**

The online version contains supplementary material available at 10.1007/s00262-023-03611-3.

## Introduction

Cancer immunotherapies aim to overcome the immune blocking mechanisms that tumors develop, reverting tumor immunosuppression and generating antitumor immunity [1]. Although cytotoxic T lymphocytes (CTLs) play a pivotal role in immune-mediated eradication of cancer [2], other immune cell subsets also demonstrated tumor-killing abilities. Among them, natural killer (NK) cells are able to discriminate tumor cells from normal cells and mediate specific antitumoral cytotoxicity [3]. Different immunotherapy strategies based on the use of NK cells are currently being explored with encouraging results [4],

The role of NK cells in cancer elimination is supported by the large amount of mechanisms that tumors evolve to inhibit NKs. For example, a large number of tumors overexpress ADAM proteases to shed MICA (MHC class I polypeptide-related sequence A) out of the cell membrane of tumor cells [5]. MICA, which is overexpressed on the surface of tumor cells due to genotoxic stress [6], is a major activator of NK cells by binding to the NKG2D receptor. By overexpressing ADAM proteases, tumor cells shed MICA, avoiding NK cell activation. Interestingly, a mutant version of MICA resistant to shedding is described [7], although its application to cancer therapy remains unexplored.

Oncolytic viruses are a novel class of immunotherapy with the capacity to both lyse cancer cells and activate antitumor immune responses [8]. Selective replication of oncolytic viruses in tumors can result in CTL responses targeting tumor antigens and overcomes, at least transiently, localized immunosuppression existing within the tumor [9]. However, oncolytic viruses tested to date have not proven efficient enough to elicit a robust antitumor immunity able to eradicate tumors. In the past, oncolytic adenoviruses have been modified to favor the antitumor effect of NKs [10, 11]. In this work, we evaluated the improvement of oncolytic adenoviral treatments by combining these vectors with the delivery of shedding-resistant MICA to activate NKs. We demonstrated that viral-mediated mutant MICA expression activates NK cells ex vivo and in mouse tumor models, and that such activation mediates improved antitumor efficacy.

## Results

### Stable expression of human MICAmut prevents tumor growth in vivo

**Fig. 1:**
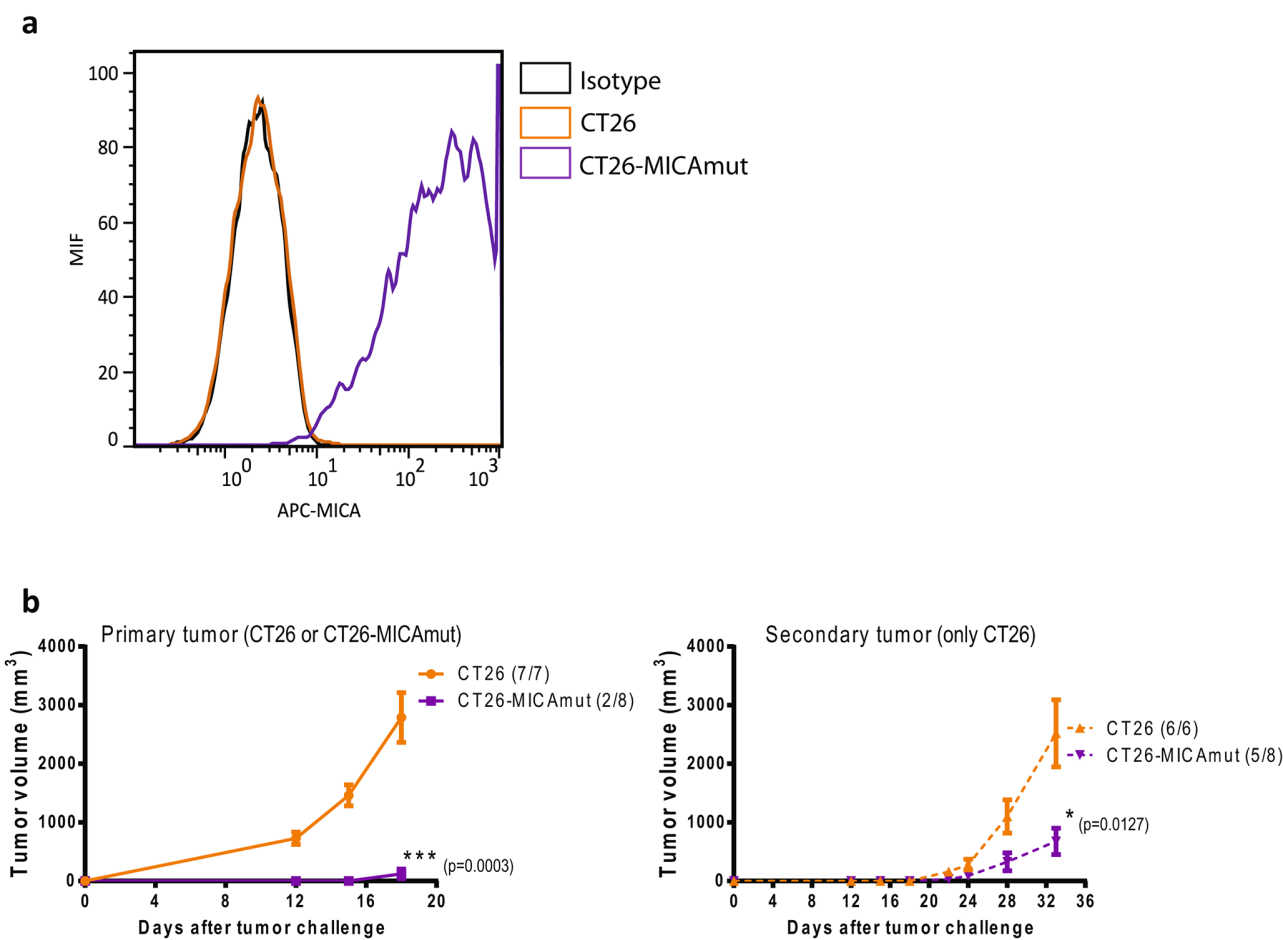
Stable expression of MICAmut by tumor cells promotes antitumor activity. (a) Determination of MICA expression by flow cytometry on CT26-MICAmut cell line. Orange and violet lines represent non-modified and MICAmut-lentiviral modified CT26 cell lines, respectively, and labelled with anti-MICA APC antibody. Black line indicates non-modified CT26 labeled with an isotype control. (b) left, tumor volume of balb/c mice injected subcutaneously with 1 × 10^6^ CT26 (orange) or CT26-MICAmut (violet) cells into the left flank; right, rechallenge performed 13 days after primary tumor implantation by injecting 1 × 10^6^ CT26 cells into the right flank of mice. The number of animals developing tumors out of the total number treated is indicated for each treatment. The mean of tumor volume ± SEM is shown. *p < 0.05, ***p < 0.001

To validate our hypothesis of the therapeutic benefit of expressing a shedding-resistant version of MICA (MICAmut) from an oncolytic adenovirus, we first evaluated the immune-stimulatory potential of stably expressing this molecule in murine tumors in vivo. For this purpose, we generated a CT26 cell line expressing MICAmut by lentiviral transduction (Fig. 1.a) and then implanted parental CT26 or CT26-MICAmut into one flank of immunocompetent mice. Thirteen days after implanting the primary tumor, a rechallenge with parental CT26 cell line was performed on the opposite flank. Analysis of tumor growth revealed an immune protective effect of MICA not only on the growth of the primary tumor, but also on the rechallenge with CT26, suggesting the role of the endogenous adaptive immune system in the observed antitumor effect (Fig. 1.b).

### Generation and characterization of an oncolytic adenovirus expressing MICAmut

**Fig. 2:**
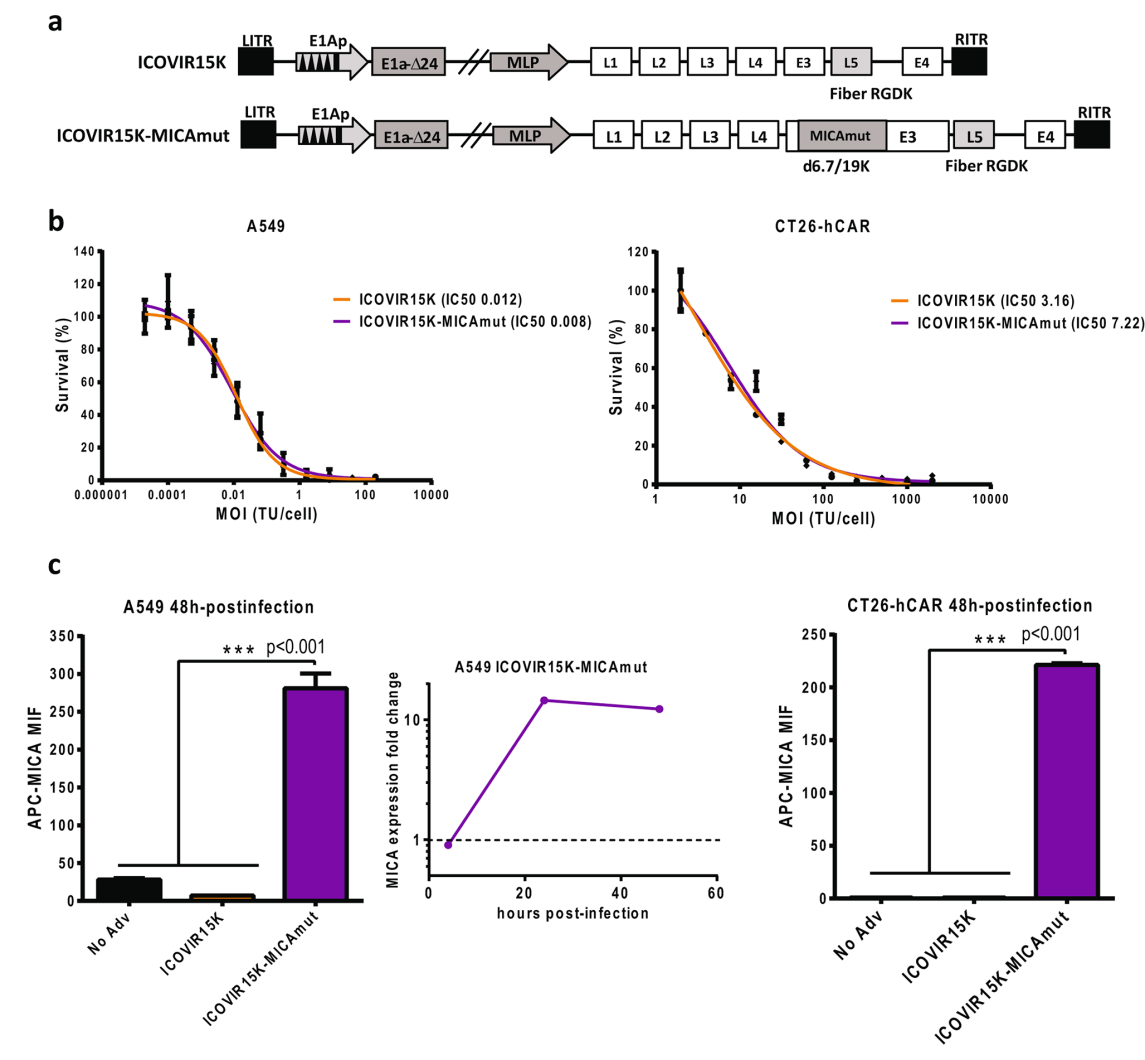
In vitro characterization of an oncolytic adenovirus expressing MICAmut. (a) Schematic representation of the genome of the viruses used in the study. Both viruses contain an E1a promoter modified by insertion of eight E2F-binding sites and one Sp1-binding site in the nucleotide 415 of the adenovirus genome; a deletion of 24 bp within the E1a region (E1A-Δ24) and the replacement of the KKTK domain in the fiber shaft with an RGD motif. ICOVIR15K-MICAmut also contains the MICA*01mut1D substituting the E3 6.7 K and gp19k adenoviral genes. (b) Cytotoxicity assay. Comparative dose-response curves of ICOVIR15K and ICOVIR15K-MICAmut at day 4 post infection in A549 (left) and CT26-hCAR (right) cells. Mean IC50 values in TU per cell for each virus is shown in the figure. (c) MICAmut expression from ICOVIR15K-MICAmut–infected cells. A549 or CT26-hCAR were infected at multiplicity of infection (MOI) of 5 or 1000, respectively, with ICOVIR15K or ICOVIR15K-MICAmut, and MICAmut surface expression was determined at 48 h post-infection by flow cytometry (left A549, right CT26-hCAR). The kinetics of MICAmut expression was also determined for infected A549 (center)

We engineered the oncolytic adenovirus ICOVIR15K [12] to express MICAmut by substituting the endogenous 6.7 K and gp19k genes (Fig. 2.a). Such an strategy has been previously used to express other transgenes [13]. Both the MICAmut-recombinant virus (ICOVIR15K-MICAmut) and its parental control are E1a-Δ24 based oncolytic adenovirus with palindromic E2F binding sites in the E1a promoter and a RGDK motif replacing the KKTK sequence in the fiber [14].

To determine whether MICAmut insertion affected viral oncolytic properties, a dose-dependent cytotoxicity assay was performed. As shown in Fig. 2.b, ICOVIR15K-MICAmut retained oncolytic properties in vitro not only in the human cell line A549, but also in the murine cell line CT26-hCAR (murine CT26 cell line modified to express the human Coxsackie and Adenovirus Receptor (CAR) in order to increase the infectivity of murine cells by human adenoviruses).

To evaluate the correct expression of MICAmut on the surface of infected cells, A549 (Fig. 2.c left) or CT26-hCAR cells (Fig. 2.c right) were infected and MICA cell surface expression density was demonstrated by flow cytometry.

### MICA expression from ICOVIR15K-MICAmut stimulates NK cells cytolytic activity

**Fig. 3:**
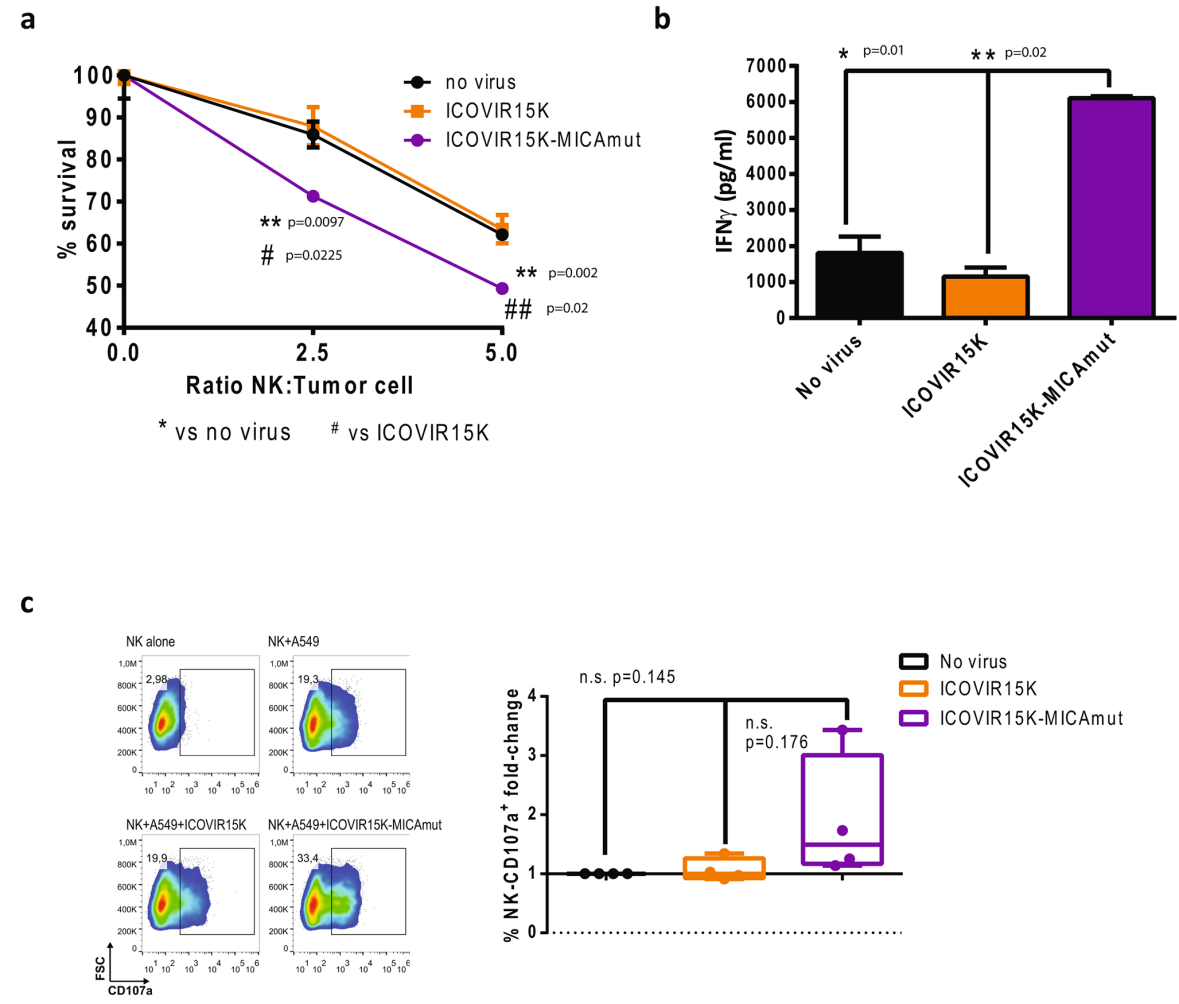
MICAmut expressed from ICOVIR15K-MICAmut–infected cells enhances NK-cell functions. NK cells were cocultured at different effector:tumor ratios with ICOVIR15K or ICOVIR15K-MICA infected A549-cGL. (a) Four hours after coculture, the survival of target cells was assessed by a bioluminescence analysis (the percentage of remaining tumor cells was calculated as (luminescence of sample/luminescence of tumor cells alone) ×100). Bars, mean ± SEM of triplicates. **p < 0.01 ICOVIR15K-MICAmut versus non-infected group; #*p* < 0.05, ##*p* < 0.01 ICOVIR15K-MICAmut versus ICOVIR15K. Two independent experiments were performed. (b) Forty-eight hours after cocultures, supernatants from the cytotoxicity assay (ratio E:T 2.5) were harvested and IFNγ production was evaluated by ELISA. Representative results from one experiment are shown. Bars, mean ± SEM of triplicates. *p<0.05; **p<0.01 ICOVIR15K-MICAmut versus non infected and ICOVIR15K groups respectively. (c) CD107a degranulation was determined by flow cytometry for NK cells cocultured during 4 h with A549 previously infected with ICOVIR15 or ICOVIR15K-MICAmut at a ratio of 4:1 effector:tumor. Left, representative example of flow cytometry CD107a degranulation analysis for each culture condition (NK cells cultured alone, with uninfected A549, and with A549 infected with each virus). NK cells were gated based on the expression of CD16 and CD56 surface markers. Right, graphical representation of the results obtained in 4 independent experiments. The CD107a expression of each group is plotted normalised to the value of the group of NKs co-cultured with uninfected A549 within each experiment

To assess whether overexpression of MICAmut in infected cells triggers enhanced NK cell-mediated cytotoxicity, we co-cultured infected or uninfected A549-GL cells (a derivative of A549 cells expressing GFP and luciferase) in the presence of human isolated NK cells at different effector:target cell ratios. After 4 h of co-culture, the antitumor activity mediated by NK cells was measured. As shown in Fig. 3.a, MICA expression significantly increased the killing capacity of NKs. This increased cytotoxic capacity was accompanied by increased IFNγ secretion (Fig. 3.b) and increased CD107a degranulation (Fig. 3.c). Altogether, these results demonstrate enhanced NK cells activity attributable to viral-mediated MICAmut overexpression on the surface of tumor cell.

### ICOVIR15K-MICAmut shows enhanced tumor growth control in vivo mediated by NK and T cells

**Fig. 4:**
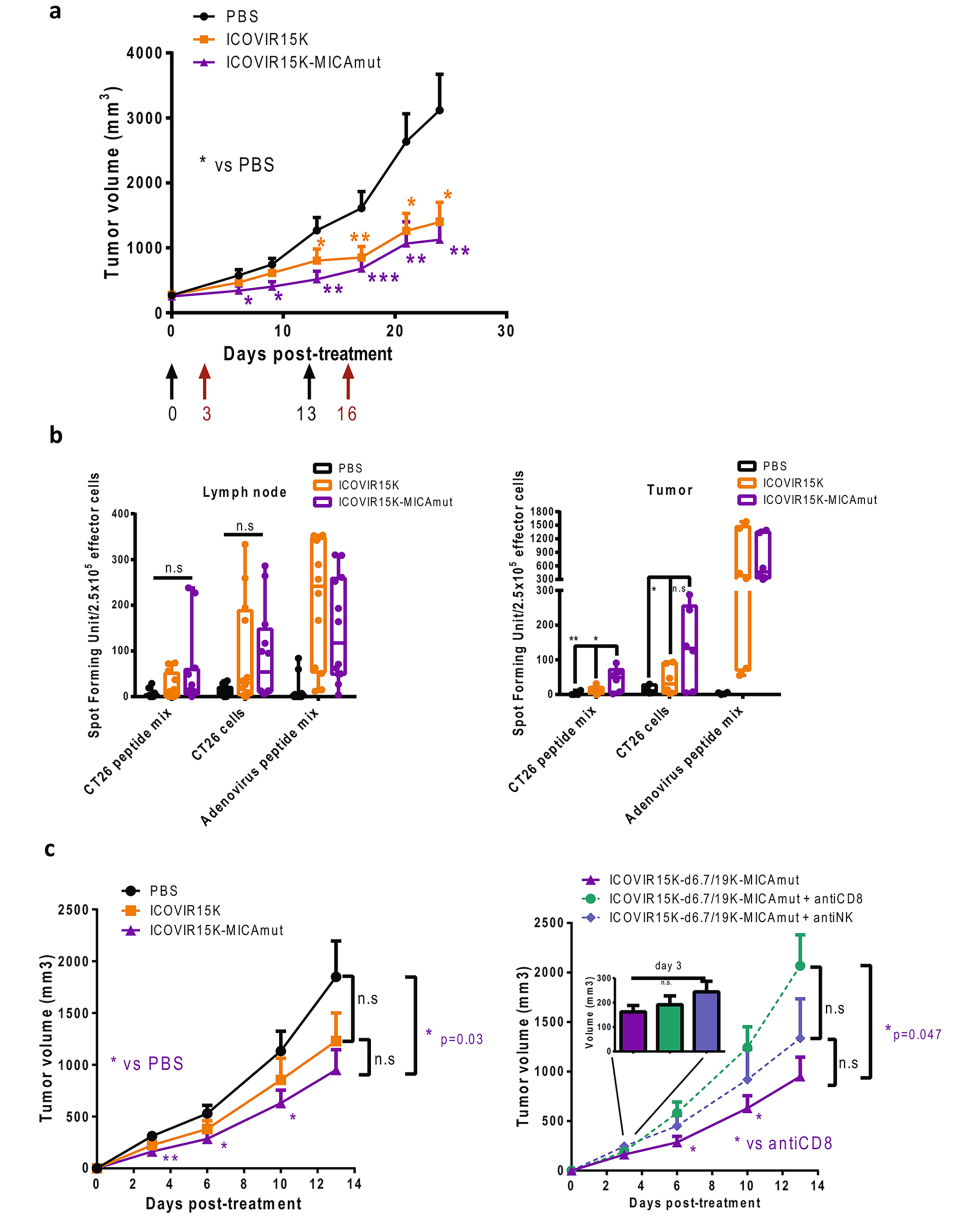
ICOVIR15K-MICAmut shows improved antitumor efficacy in vivo. (a) NSG mice bearing subcutaneous A549 tumors were intratumorally injected with PBS or ICOVIR15K or ICOVIR15K-MICAmut twice, on days 0 and 13. Three days after each virus administration, mice received an intravenous injection of 1 × 10^7^ human PBMCs and tumor volume was periodically determined. The mean tumor volume ± SEM is shown. *p < 0.05, **p < 0.01; ***p < 0.001 ICOVIR15K-MICAmut and ICOVIR15K versus PBS group. (b) Balb/c mice bearing subcutaneous CT26-hCAR tumors were intratumorally injected with PBS, ICOVIR15K, or ICOVIR15K-MICAmut (1 × 10^9^ TU/tumor) on days 0, 3 and 6. On day 11, mice were sacrificed and an IFN-γ ELISpot assay were performed on single-cell suspensions from lymph nodes (left) or tumors (right). Mouse lymphocytes were stimulated overnight with a mix of CT26 specific neopitopes peptides, whole CT26 cells at a ratio 1:1 or and adenovirus mix epitopes peptides in duplicates. Individual values of IFN-γ spot forming units/2.5 × 10^5^ cells in 6 mice/group and means ± SD are plotted on the graphs. *p < 0.05, **p < 0.01 ICOVIR15K-MICAmut versus PBS and ICOVIR15K groups. (c) Left, the same animal model and treatment approach described in b) was carried out to evaluate the antitumor activity of ICOVIR15K-MICAmut. PBS injected mice were used as the control group. To monitor tumor volume, tumors were measured 2–3 times per week. The mean tumor volume ± SEM is shown. *p < 0.05, **p < 0.01 ICOVIR15K-MICAmut versus PBS group. Right, to evaluate the relevance of CD8 + T cells and NKs cells in the antitumor efficacy observed for ICOVIR15K-MICA, balb/c mice with subcutaneous CT26-hCAR tumors were injected with CD8 or NK-depleting antibodies and treated as before. Again, tumors were measured 2–3 times per week. The mean tumor volume ± SEM is shown. *p < 0.05 ICOVIR15K-MICAmut non depleted versus ICOVIR15K-MICAmut CD8-depleted group. A zoom on day 3 is shown to reflect the impact of NK cells depletion at an early time point

Two different mouse model have been evaluated in this study. First, we wanted to determine whether human NK cells stimulation observed in vitro translates into enhanced anti-tumor efficacy in vivo. To address this hypothesis, immunodeficient NSG mice bearing subcutaneous human A549 tumors were treated intratumorally twice with ICOVIR15K-MICAmut or controls on days 0 and 13. Three days after viral treatments, mice received intravenous injections of 10^7^ human PBMCs (on days 3 and 16). As indicated in Fig. 4.a, MICA-expressing virus shows improved tumor growth control capacity compared to the parental virus throughout the study, although the difference was not significant.

Next, we wanted to evaluate ICOVIR15K-MICAmut antitumor efficacy in an Balb/c immunocompetent model. Mice bearing CT26-hCAR tumors were intratumorally injected (thrice, on days 0, 3 and 6) with ICOVIR15K-MICAmut, the control virus, or PBS. Five days after the last administration, anti-tumor and anti-viral immune responses were evaluated by ELISpot in immune cells isolated from lymph nodes and tumors (Fig. 4.b). To determine anti-tumor immunities, both the whole tumor cell and T cell tumor-specific epitopes were used as targets. A strong antiviral T-cell response was observed in lymphocytes isolated from both lymph nodes and tumors independently of MICAmut expression. However, ICOVIR15K-MICAmut significantly increased the tumor-infiltrating lymphocytes reactivity against the whole tumor cell and T cell tumor-specific epitopes, an increase not observed in lymphocytes isolated from lymph nodes. To assess whether this anti-tumor immune-stimulation led to improved anti-tumor efficacy, Balb/c mice bearing CT26-hCAR tumors were treated as previously and tumor volume was monitored. As observed in Fig. 4.c left, only the treatment with ICOVIR15K-MICAmut showed a significant ability to control tumor growth compared to PBS. Finally, to further demonstrate that the enhanced antitumor activity of MICAmut-expressing virus is immune-mediated, we evaluated whether depletion of CD8 + T cells or NK cells has an impact on antitumor efficacy. The analysis of the tumor growth kinetics for the different groups seems to indicate that NK cells at early time points (day 3) and CD8 + T cells at later ones appeared responsible for such an improvement as their depletion translated into a loss of ICOVIR15K-MICAmut-mediated antitumor efficacy (Fig. 4.c right).

Altogether, our results highlight the relevance of expressing a shedding-resistant MICA to improve current viral immunotherapies.

## Discussion

The role of NK cells in tumor eradication is generally underestimated. However, NK cells are capable of killing cancer cells without activation by antigen-presenting cells [15] and patients who suffer NK cell dysfunction have increased rates of malignancies [16]. In addition, previous reports demonstrated that the contribution of NK cells to the antitumor efficacy of viral immunotherapies is comparable to that of CTLs [17]. As NKG2D engagement is sufficient by itself to activate and degranulate NK cells [18], in this work we evaluated the antitumor effects of this engagement via a viral delivery of an NKG2D ligand. Although NKG2D ligands significantly vary within species [19], the fact that the human ligand MICA is recognized by mouse NKG2D and activates mouse NK cells [20] allows us to evaluate our strategy in tumor mouse models. Importantly, the application for this therapy of a shedding-resistant version of MICA previously described [7] overcomes the mechanisms that tumors evolve to shed MICA from the surface of tumor cells [5]. In addition, this mutant version is based on the MICA*001 allele, which is described to have one of the highest affinities for NKG2D [21]. Although MICA is highly polymorphic within the human population [22], our therapy would not be restricted to patients with MICA*001 allele as NKG2D from patients with other alleles would still be activated by our expressed molecule.

Our results indicate that our strategy holds great promise for the treatment of solid tumors. First, we generated a mouse tumor cell line stably expressing MICAmut as a proof-of-concept, and our results demonstrated that MICAmut expression by cancer cells leads to robust antitumor effects and antitumor immunity. Next, we chose an oncolytic adenoviral vector to deliver MICAmut. This virus demonstrated to efficiently express MICAmut after infection of cancer cells and such an expression did not affect the oncolytic properties of the vector. Viral-mediated MICAmut expression in cancer cells activated human NK cells ex vivo and mediated improved antitumor effects of injected human or endogenous murine immune cells in mouse tumor models. Importantly, significant increased numbers of endogenous murine immune cells reactive to tumor cells infiltrating the tumors were detected. The number of cells reacting to T cell tumor-specific epitopes was lower than the number of cells reacting to the whole tumor cells as measured by interferon-γ ELISpot; probably because of T cells responding against tumor epitopes not included in the tumor peptides mix, but also due to NK cells infiltrating the tumor. In addition, NKG2D is also expressed on NKT cells, γδ T cells, and activated CD8 + T cells [23], which could also contribute to the outcome of our therapy. In fact, depletion of both endogenous murine CD8 + and NK cells demonstrated to play an important role in the outcome of the therapy as their depletion leads to reduced antitumor efficacy.

Although the benefits observed with our oncolytic adenovirus expressing MICAmut, we think that the antitumor potential of our approach is underestimated by the tumor models available. Two different mouse tumor models were chosen in this study: the first one consisted on administrating human PBMCs to immunodeficient mice harboring human tumors. In this model, the oncolytic properties of the vector are prioritized but the antitumor effects of administrated PBMCs (including NK cells) are very limited. The second model consisted syngeneic mouse tumor cell lines implanted into immunocompetent mice. Although the tumor cell line was modified to express human CAR to improve infectivity, human adenovirus replication in mouse cells is very limited [24]. Thus, the viral-mediated lysis and amplification of the initial viral dose administrated, which would result in magnification of MICAmut expression, are not evaluated in this setting. Thus, the real potential of our approach would only be apparent in human immunocompetent individuals, such as in the clinical setting.

In summary, our work not only describes the immune-stimulatory properties and antitumor potential of ICOVIR15K-MICAmut, but also pointed out its potential as candidate to combinate with immunotherapies based on adoptive transfer of unmodified or modified NK cells, such as NK-CAR or NK-TcR cells in which MICA expressed from ICOVIR15K-MICAmut would increase the infiltration of NK cells into the tumors and their subsequent activation.

## Conclusion

Viral-mediated expression of a shedding-resistant NKG2D ligand activates NK cells ex vivo and results in improved antitumor immune responses in mouse tumor models.

## Materials and methods

### Cell lines and viruses

The cell lines A549, CT26, and HEK293 were purchased from the American Type Culture Collection. The A549-GL cell line was generated using a lentiviral vector encoding for GFP and luciferase. CT26-hCAR and CT26-MICAmut were produced by infecting with lentiviral vectors encoding for the human Coxsackie and Adenovirus Receptor or the MICA*01mut1D gene [7], respectively. Cell lines were cultured in recommended culture media containing 10% fetal bovine serum (FBS) and antibiotics at 37ºC, 5% CO_2_.

PBMCs from healthy donors were isolated from blood by ficoll density gradient centrifugation and cultured in complete RPMI-1640 10% FBS. NK cells were isolated from total PBMCs using human CD56 microbeads, LS columns, and MidiMACS separator (Miltenyi Biotec), and cultured with NK MACs medium (Miltenyi Biotec) with 5% human AB serum (Biowest) and 200 IU/ml of IL2 (Clinigen).

### Recombinant adenovirus

ICOVIR15K was previously described [12]. ICOVIR15K-MICAmut was generated by homologous recombination in bacteria by substituting the E3 6.7 K and gp19k genes for the MICA*01mut1D gene in a plasmid including the whole ICOVIR15K genome. The ICOVIR15K-MICAmut plasmid was transfected into HEK293 cells, and the generated virus was amplified in A549 cells and purified on a CsCl gradient according to standard protocols.

### Virus cytotoxicity assays

Virus cytotoxicity assays were performed as previously described [12], 4 days post-infection. IC50 was calculated using GraphPad Prism v6.02.

### Kinetics of MICAmut expression in infected cells

A549 or CT26-hCAR were infected at multiplicity of infection (MOI) of 5 or 1000, respectively, with ICOVIR15K or ICOVIR15K-MICAmut. After 4, 24, and 48 h, cells were harvested, incubated with APC-labelled anti-human MICA/MICB antibody (Biolegend) and analyzed using a Gallios Cytometer (Beckman Coulter).

### In vitro cytotoxicity assay by NK cells and interferon-γ quantification

A549-cGL were infected with ICOVIR15K or ICOVIR15K-MICAmut at MOI 5 and, 24 h after infection, human NK cells and infected tumor cells were incubated together at different effector:tumor cells ratios for 4 h. Bioluminescence emitting from live cells was determined on a Victor X reader (Perkin Elmer).

In parallel, supernatant from cytotoxicity assays were collected and human IFNγ levels were assessed using an ELISA kits (BioLegend), according to the manufacturer’s instructions.

### NK cells CD107a degranulation assay

A549 were infected with ICOVIR15K or ICOVIR15K-MICAmut at MOI 5 and, 24 h later, co-cultured with human NK cells at a ratio of 4:1 effector:tumor cells. Immediately after, antihuman BV-421-labelled CD107a antibody (BD Biosciences) was added and, after 1 h of incubation, a protein transport inhibitor (BD GolgiStop) was also added. Finally, after 4 h of culture, samples were stained with APC-anti human CD56 and PE-anti human CD16 and analyzed by flow cytometry.

### Mouse models

For the rechallenge experiment, Balb/C mice were injected subcutaneously with 1 × 10^6^ CT26 or CT26-MICAmut cells into the left flank. Rechallenge was performed 13 days after primary tumor implantation by injecting 1 × 10^6^ CT26 cells into the right flank of mice. Tumor volume was periodically determined by caliper measures.

In another setting, CT26-hCAR (1 × 10^6^ cells) were subcutaneously injected into the flank of Balb/c mice and, when tumors reached a volume of 50–100 mm^3^, viruses were administrated intratumorally at a dose of 1 × 10^9^ TU/tumor on days 0, 3 and 6 and tumor volume was periodically calculated. For the depletion experiment, mice were injected intraperitoneally with 250 µg of rat IgG2b isotype control or anti-mouse CD8α antibodies (BioXCell), or 50ul of anti-Asialo-GM1 antibody (Fujifilm Wako) the day before the first viral administration (day − 1), followed by 100 µg or 20 µl at days 2 and 7 for isotype control and anti-CD8α or 3 and 8 for anti-Asialo-GM1.

Human lung adenocarcinoma xenograft tumors were established by implanting 5 × 10^6^ A549 cells subcutaneously into both flanks of NOD/SCID gamma (NSG) mice. When tumors reached 100–120 mm^3^, mice were injected twice (days 0 and 13) with an intratumoral dose of 3 × 10^8^ TU of indicated viruses. On days 3 and 16, 1 × 10^7^ human PBMCs were administered to the mice by intravenous injection and tumor volume was periodically determined.

### IFN-γ ELISpot

IFN-γ ELISpot assays were performed on single-cell suspensions from lymph nodes or tumors of Balb/C mice treated with PBS or the different viruses (specific protocols and common reagents used for isolation in [25]). Samples were obtained from Balb/C mice treated with 3 intratumor administrations of indicated viruses as previously indicated (on days 0, 3 and 6) and sacrificed on day 11. Mouse lymphocytes were stimulated overnight with: 1- CT26 synthetic specific peptides (based on previously described and validated CT26 neopitopes SmC3 (KFKASRASI), ME1 (HSGQNHLKEMAISVLEARACAAAGQ), AH1 (SPSYVYHQF), MO3 (KPLRRNNSYTSYIMAICGMPLDSFR), M37 (VIQTSKYYMRDVIAIESAWLLELAP), M26 (ILPQAPSGPSYATYLQPAQAQMLTP), 23 (SWIHCWKYLSVQSSQLFRGSSLLFRR) [26–29]); 2- Adenovirus mix epitopes peptides (Hex3 (KYSPSNVKI) and DBP7 (LPKLTPFAL)); or 3- whole CT26 cells at a ratio 1:1.

### Statistical analysis

Statistical comparisons between two groups were performed using the Mann–Whitney U test. For comparison of more than two groups, Kruskal–Wallis with Dunn post hoc test was used. Statistical significance was established as *p* < 0.05. Data are presented as the mean ± SD or SEM. All statistical analysis were calculated with GraphPad Prism software.

### Electronic supplementary material

Below is the link to the electronic supplementary material.

Supplementary Material 1

## Data Availability

Data are available upon request. All data relevant to the study are included in the article.
