# Supplementary material for: Oncolytic adenovirus coding for shedding-resistant MICA enhances immune responses against tumors
Source: Cancer Immunol Immunother. 2024 Jan 5;73(1):5. doi: 10.1007/s00262-023-03611-3 (PMC10770194; doi:10.1007/s00262-023-03611-3)
Supplement: Supplementary file 1 — Supplementary Material 1 [file 262_2023_3611_MOESM1_ESM.docx]

**Material and methods**

**Cell lines and viruses**

The cancer cell lines A549 (human lung adenocarcinoma) 293 (human kidney (embryonic)) and CT26 (BALB/c mouse undifferentiated colon carcinoma) were obtained from the American Type Culture Collection (ATCC, Manassas, VA).  The A549-GL cell line was generated using a lentiviral vector encoding GFP and luciferase. CT26-hCAR was generated by sorting CT26 cells previously transduced with a lentiviral vector encoding the human Coxsackievirus and adenovirus receptor (CAR) (GenBank: AAC51234.1). Similarly, the CT26-MICAmut were generated modifying the original CT26 with a lentiviral vector encoding the MICA*01mut1D gene, a human MHC class I chain-related polypeptide A (MICA) mutant resistant to proteolysis by ADAM proteases (kindly provided by Dr. Alexander Steinle) [7]. A549 cell lines was cultured in Dulbecco's Modified Eagle medium complete and CT26 in RPMI-1640, supplemented with 10% fetal bovine serum, 2 mM L-glutamine, 1% (v/v) penicillin/streptomycin (all from Thermo Fisher Scientific, Waltham, MA, USA) and maintained at 37°C in 5% CO2. Cell lines were routinely tested for mycoplasma presence.

Experiments employing human PBMCs were approved by the ethics committees of the University Hospital of Bellvitge and the Blood and Tissue Bank from Catalonia. PBMCs of healthy donors were isolated from blood by ficoll (Rafer, Spain) density gradient centrifugation in Leucosep tubes (Greiner Bio-one, Kremsmünster, Austria) following the manufacturer’s recommendations and cultured in RPMI-1640, supplemented with 10% fetal bovine serum, 2 mM L-glutamine, 1% (v/v) penicillin/streptomycin. When used, NK cells were isolated from total PBMCs using human CD56 microbeads, LS columns, and MidiMACS separator (all from Miltenyi Biotec, Gladbach, Germany), and culture with NK MACs medium (Miltenyi Biotec) supplemented with 5% human AB serum (Biowest, Nuaillé, France), 1% (v/v) penicillin/streptomycin (Thermo Fisher Scientific) and 200IU/ml of IL2 (proleukin, Clinigen, London, UK).

**Recombinant Adenovirus**

ICOVIR15K were previously described [10]. ICOVIR15K-d6.7/19K-MICAmut was generated by homologous recombination in bacteria as described [13, 14]. First, we replaced the E3 6.7K and gp19k genes by the rpsLNeo selection cassette. This step generated the 304-323 deletion previously described for ONYX-304 [12]. In a second step, we replace the selection cassette by the gene MICA*01mut1D. The ICOVIR15K-d6.7/19K-MICAmut plasmid was transfected into HEK293 cells, and the resulting virus was amplified in successive rounds in A549 cells and purified on a CsCl gradient according to standard protocols.

**Virus cytotoxicity assays**

Virus cytotoxicity assays were performed as previously described [10]. Briefly, a serial dilution of viral TU was used to infect A549d cell line in triplicate. The initial multiplicity of infection (MOI; TU/cell) and the number of cells should be carefully adjusted depending on the cell line. After 4 days of infection, the cell viability was assessed by bicinchoninic acid assay (BCA, Pierce Biotechnology). Absorbance was quantified, and the number of TU per cell required for 50% inhibition (IC50) was estimated from a dose-response non-linear regression with a variable slope, calculated with GraphPad Prism v6.02 (GraphPad Software).

**Kinetics of mica expression in infected cells**

A549 or CT26-hCAR were infected at multiplicity of infection (MOI) of 5 or 1000, respectively, with ICOVIR15K or ICOVIR15K-d6.7/19K-MICAmut. After 48h, cells were harvested with versene solution (Thermo Fisher Scientific), incubated with APC-labelled anti-human MICA/MICB (Biolegend, San Diego, CA, USA) for 30min at 4ºC. A Gallios Cytometer (Beckman Coulter) was used, and 1× 10^4^ events were analyzed for each sample. FlowJo v7.6.5 (Tree Star, Inc.) software was used for the analysis of the data. For the determination of the kinetics of MICA expression, A549 cells were seeded in 12-well plates (1× 10^5^ cells/well) and infected with ICOVIR15K-d6.7/19K-MICAmut at MOI 5. MICA expression was determined as previously described at 4, 24 and 48h post-infection. MICA basal expression in non-infected A549 cells was also analysed. MICA overexpression from infected cells is expressed as fold-change respect non-infected cells.

***In vitro* cytotoxicity assay**

A549-cGL were seeded in 6-well plates (4× 10^5^ cells/well) and infected with ICOVIR15 or ICOVIR15K-d6.7/19K-MICAmut at MOI 5. In parallel, NKs cells were isolated from fresh blood and cultured in the presence of 200IU/ml of IL2. After 24h, NK cells and tumoral cells were incubated in triplicates at several effector:tumor ratios in 96-well U-bottom plates for 4h at 37ºC and 5%CO_2_. Then, samples were transferred to white-walled 96-well and D-Luciferin (20mg/ml) added. After 15min, bioluminescence was determined on a Victor X reader (Perkin Elmer, Waltham, MA, USA). The percentage of remaining tumor cells was calculated as (luminescence of sample/luminescence of tumor cells alone) ×100.

**ELISA**

Supernatant from cytotoxicity assays were collected and human IFNγ level was assessed by ELISA kits (BioLegend), according to the manufacturer's instructions.

**NK cells CD107a degranulation assay**

The day before the experiments, NKs were isolated from peripheral blood and culture in the presence of 200IU/ml of IL2, and A549 were seeded in 12-well plates (1.5× 10^5^ cells/well) and infected with ICOVIR15 or ICOVIR15K-d6.7/19K-MICAmut at MOI 5. 24h later, 1x10^5^ target cells and 4x10^5^ NK cells were co-cultured in a U-bottom 96-well culture plate. Right afterward, antihuman BV-421-labelled CD107a antibody (BD Biosciences, San Jose, CA, USA) were added to each well and incubate for 4 h at 37 °C and 5% CO2. After 1 h of incubation, a protein transport inhibitor (BD GolgiStop) was also added. Finally, after 4h of culture, samples were stained with APC-anti human CD56 and PE-anti human CD16 and analyzed by flow cytometer.

**In vivo studies**

In vivo studies were performed at the ICO-IDIBELL animal facility (Barcelona, Spain) AAALAC unit 1155 and approved by IDIBELL’s Ethical Committee for Animal Experimentation.

*Immunocompetent mice.*

Rechallenge experiment. Subcutaneous CT26 or CT26-MICAmut tumors were established by injection of 1x10^6^ cells into the left flank of 8-week-old female Balb/c mice (Charles River Laboratories). The rechallenge was performed 13 days after implantation of the primary tumour by injecting 1x10E6 CT26 into the right flank of the mouse. Tumor volume was periodically calculated according to equation V (mm3) = π/6 × W^2^ × L, where W and L are the width and the length of the tumor, respectively.

Antitumor efficacy.

CT26-hCAR (1x10^6^ cells) were subcutaneously injected into the flank of 8-week-old female Balb/c mice. When tumors reached a volume of 50–100 mm^3^, mice were randomized, and viruses were administrated intratumorally (1x10^9^ TU/tumor, on day 0, 3 and 6). Tumor volume was periodically calculated as previously. Tumor growth, expressed as the tumor size relative to the size at the beginning of the therapy, was also determined. A second experiment was performed as indicated but animals were sacrificed at day 10 for specific antitumor immune response analysis by ELISPOT. Immune cells from lymph nodes and tumors were isolated as described [15].

For depletion experiments, anti-mouse CD8a (2.43, Ref BE0061) was purchased from BioXCell (Lebanon, NH, USA), and anti-mouse Asialo-GM1 was purchased from Fujifilm Wako Pure Chemicals (Osaka, Japan). Mice were injected intraperitoneally with 250 µg of anti-CD8α or 50ul of anti-NKs antibodies the day before the first viral administration (day -1), followed by 100μg or 20μl at days 2 and 7 for anti-CD8α or 3 and 8 for anti-NKs.

*Immunodeficient mice.*

Lung adenocarcinoma xenograft tumors were established by implanting 5 × 10^6^ A549 cells subcutaneously into both flanks of 8-week-old NOD/SCID gamma (NSG) mice. When tumors reached 100–120 mm3, mice were randomized and distributed into groups. Mice were injected twice (days 0 and 13) with and intratumoral dose of 3x10^8^ TU. On days 3 and 16, 1x10^7^ human PBMCs were administered to the mice by intravenous injection. Tumor volume and growth were determined.

**IFN-γ ELISpot**

IFN-γ ELISpot assays were performed on single-cell suspensions of lymph nodes or tumor of Balb/C mice treated with PBS or the different viruses according to standar protocols (specific protocol and common reagents details in [16]). Mouse lymphocytes were plated in duplicate for each condition, and stimulated overnight with: CT26 synthetic specific peptides (based on previously described and validated CT26 neopitopes SmC3 (KFKASRASI), ME1 (HSGQNHLKEMAISVLEARACAAAGQ), AH1 (SPSYVYHQF), MO3 (KPLRRNNSYTSYIMAICGMPLDSFR), M37 (VIQTSKYYMRDVIAIESAWLLELAP), M26 (ILPQAPSGPSYATYLQPAQAQMLTP), 23 (SWIHCWKYLSVQSSQLFRGSSLLFRR) [17–20]); adenovirus mix epitopes peptides (Hex3 (KYSPSNVKI) and DBP7 (LPKLTPFAL)); CT26 cells at a ratio 1:1; and medium or PMA/ionomicyn as negative and positive controls respectively.

**Statistical analysis**

Statistical comparisons between two groups were performed using the Mann–Whitney U test. For comparison of more than two groups, Kruskal–Wallis with Dunn post hoc test was used. Statistical significance was established as p < 0.05. Data are presented as the mean ± SD or SEM. All statistical analysis were calculated with GraphPad Prism software.
